# Enhanced and unenhanced: Radiomics models for discriminating between benign and malignant cystic renal masses on CT images: A multi-center study

**DOI:** 10.1371/journal.pone.0292110

**Published:** 2023-09-28

**Authors:** Lesheng Huang, Wenhui Feng, Wenxiang Lin, Jun Chen, Se Peng, Xiaohua Du, Xiaodan Li, Tianzhu Liu, Yongsong Ye

**Affiliations:** 1 Department of Radiology, Guangdong Provincial Hospital of Traditional Chinese Medicine, Zhuhai, China; 2 Department of Radiology, Zhuhai People’s Hospital, Zhuhai, China; 3 Department of Laboratory, Guangdong Provincial Hospital of Traditional Chinese Medicine, Zhuhai, China; 4 Department of Gynaecology, Guangdong Provincial Hospital of Traditional Chinese Medicine, Zhuhai, China; Hokkaido University: Hokkaido Daigaku, JAPAN

## Abstract

**Background:**

Machine learning algorithms used to classify cystic renal masses (CRMs) nave not been applied to unenhanced CT images, and their diagnostic accuracy had not been compared against radiologists.

**Method:**

This retrospective study aimed to develop radiomics models that discriminate between benign and malignant CRMs in a triple phase computed tomography (CT) protocol and compare the diagnostic accuracy of the radiomics approach with experienced radiologists. Predictive models were established using a training set and validation set of unenhanced and enhanced (arterial phase [AP] and venous phase [VP]) CT images of benign and malignant CRMs. The diagnostic capabilities of the models and experienced radiologists were compared using Receiver Operating Characteristic (ROC) curves.

**Results:**

On unenhanced, AP and VP CT images in the validation set, the AUC, specificity, sensitivity and accuracy for discriminating between benign and malignant CRMs were 90.0 (95%CI: 81–98%), 90.0%, 90.5% and 90.2%; 93.0% (95%CI: 86–99%), 86.7%, 95.2% and 88.3%; and 95.0% (95%CI: 90%-100%), 93.3%, 90.5% and 92.1%, respectively, for the radiomics models. Diagnostic accuracy of the radiomics models differed significantly on unenhanced images in the training set vs. each radiologist (p = 0.001 and 0.003) but not in the validation set (p = 0.230 and 0.590); differed significantly on AP images in the validation set vs. each radiologist (p = 0.007 and 0.007) but not in the training set (p = 0.663 and 0.663); and there were no differences on VP images in the training or validation sets vs. each radiologist (training set: p = 0.453 and 0.051, validation set: p = 0.236 and 0.786).

**Conclusions:**

Radiomics models may have clinical utility for discriminating between benign and malignant CRMs on unenhanced and enhanced CT images. The performance of the radiomics model on unenhanced CT images was similar to experienced radiologists, implying it has potential as a screening and diagnostic tool for CRMs.

## Introduction

Cystic renal masses (CRMs) are lesions with less than 25% enhancing tissue that are often identified incidentally on abdominal computed tomography (CT) scans [1]. The majority of CRMs are benign, but some may be renal cell carcinoma (RCC) or other rare malignant tumors of the kidney [2, 3]. The 2019 Bosniak classification of CRMs, updated from 2005, stratifies CRMs according to their risk of malignancy [1]. In individual series, most Bosniak I and II CRMs are benign, while approximately 10–20% of Bosniak IIF, 50% of Bosniak III, and 90% of Bosniak IV CRMs are malignant [3, 4]. The variable malignancy risk in Bosniak IIF and III CRMs has a substantial psychological impact on patients, and necessitates follow-up, which incurs significant health care resource utilization and costs [5–7].

There is an unmet clinical need for an objective strategy that assists radiologists and surgeons in the identification of benign and malignant CRMs on CT images. The Bosniak classification has inherent limitations such as bias and inter-observer variability, despite clearly defined terms, imaging features, and classes [5–8]. Radiologists rely on visual inspection of CT images for the diagnosis of CRMs, which can be impacted by image noise and resolution, especially on unenhanced scans [4].

Emerging research shows that radiomics features can reveal key components of tumor phenotype in three-dimensions [9], and radiomics models have excellent diagnostic efficacy for RCC on enhanced and unenhanced CT scans [10–14]. Several machine learning algorithms have been applied to classify CRMs as benign or malignant on contrast-enhanced CT images taken in the arterial phase (AP) and venous phase (VP) [15–17]; however, these algorithms were not applied to unenhanced CT images alone or validated with external datasets [15], and their diagnostic accuracy was not compared against radiologists. The objective of this multicenter study was to use a radiomics approach to discriminate between benign and malignant CRMs on all phases of a triple phase CT protocol (unenhanced, AP, VP) and compare the diagnostic accuracy of the radiomics approach with experienced radiologists.

## Materials and methods

### Patients

This retrospective study was approved by the medical ethics committee of the Guangdong Provincial Hospital of Traditional Chinese Medicine (No. ZE2023-090-01), and the requirement for written informed consent was waived.

Patients with CRMs who were treated at Guangdong Provincial Hospital of Traditional Chinese Medicine, Guangzhou and Guangdong Provincial Hospital of Traditional Chinese Medicine, Zhuhai between January 2018 and June 2023 were eligible for this study. Inclusion criteria were: 1) ≥ Bosniak category IIF CRMs; 2) unenhanced and enhanced CT scans (including AP and VP images); 3) complete records of patient demographic and clinical characteristics, including age, sex, location of masses, intraoperative or biopsy results and histopathological findings; and 4) CT images stored in the picture archiving and communication system (PACS) of the institution. Patients with low-quality or incomplete CT data were excluded.

### CT examinations

All patients underwent unenhanced and dual-phase contrast-enhanced CT using three CT scanners (Definition flash, Siemens, Forchheim, Germany/ in Guangzhou; IQon Spectral, Philips Healthcare, Amsterdam, Netherlands/ in Guangzhou; Aquilion One 750W, Canon, Tokyo, Japan/ in Zhuhai). Imaging parameters were: tube voltage 120 kV, tube current 250 mA, section interval 5 mm, section thickness 5 mm, and matrix 512 mm × 512 mm. After conventional unenhanced imaging, 100–120 ml iopromide (Ultravist 370, Bayer Schering Pharma, Germany) was injected into the median cubital vein via a pump injector (MEDRADStellant CT, Ulrich Medical, Ulm, Germany) at 3–4 ml/s. The AP scan was triggered by aortic enhancement. The VP scan started at a delay of 60 s from the beginning of contrast injection.

One radiologist (LH) with 18 years of experience analyzed the CT images to ensure the renal masses had < 25% enhancing tissue (i.e. lesions were cystic masses), confirm the Bosniak class (Version 2019), and determine the size of the CRMs.

### Mass segmentation and radiomics feature extraction

The open-source software platform, 3D-slicer version 5.2.1 (www.slicer.org) was used for mass segmentation and to calculate radiomics features. Masses were delineated on the CT images using 3D-slicer. Segmentation of whole masses was performed by associate chief radiologists (LT and HL), who have more than fifteen years of experience with abdominal radiographs. After segmentation, 855 radiomics features were extracted using the Python pyradiomics package (JetBrains PyCharm Community Edition 2017.2.4; https://www.jetbrains.com/pycharm/). To ensure the stability of radiomics features extracted from CT images, segmentation and feature extraction were repeated in 80 randomly selected patients with CRMs. The intraclass correlation coefficient (ICC) was used to evaluate consistency across radiomics features. Radiomics features with ICC > 0.75 were considered stable and were included in this analysis.

### Diagnostic efficacy of radiologists

The diagnostic efficacy of radiologists was assessed in two attending radiologists (HF and XL) with more than seven years of experience in diagnostic imaging who used the open-source DICOM viewer, MicroDicom (https://www.microdicom.com/). The radiologists were from hospitals not involved in this study and were blinded to patient demographic and clinical characteristics. The radiologists independently reviewed the CT images in the following order, unenhanced, AP and VP, with a two-week interval between each phase. Diagnosis was based on the Bosniak classification and the radiologists’ clinical experience.

### Statistical analysis

Statistical analysis was conducted with SPSS (version 26.0, IBM, Armonk, NY, USA) and R (version 4.2.2). A two-sided *P* value <0.05 was considered statistically significant.

Differences in demographic and clinical characteristics were assessed using the *x*^*2*^ test or independent sample t-test, as appropriate.

Radiomics models were established using a training set of 77 benign and 85 malignant CRMs from Guangdong Provincial Hospital of Traditional Chinese Medicine, Guangzhou. The models were tested with a validation set of 15 benign and 30 malignant CRMs from Guangdong Provincial Hospital of Traditional Chinese Medicine, Zhuhai.

Radiomics feature selection was necessary to avoid overfitting the models. Univariate analysis (Student’s t-test or Mann–Whitney U test) was used to extract stable radiomics features that were statistically different between benign and malignant CRMs in the training set. Candidate radiomics features to generate the models were selected with the least absolute shrinkage and selection operator (LASSO) using the “glmnet” package in R. Bidirectional elimination was used to filter out potentially irrelevant radiomics features using the “mass” package in R. Selected features were combined into linear regression equations.

**Fig 1:**
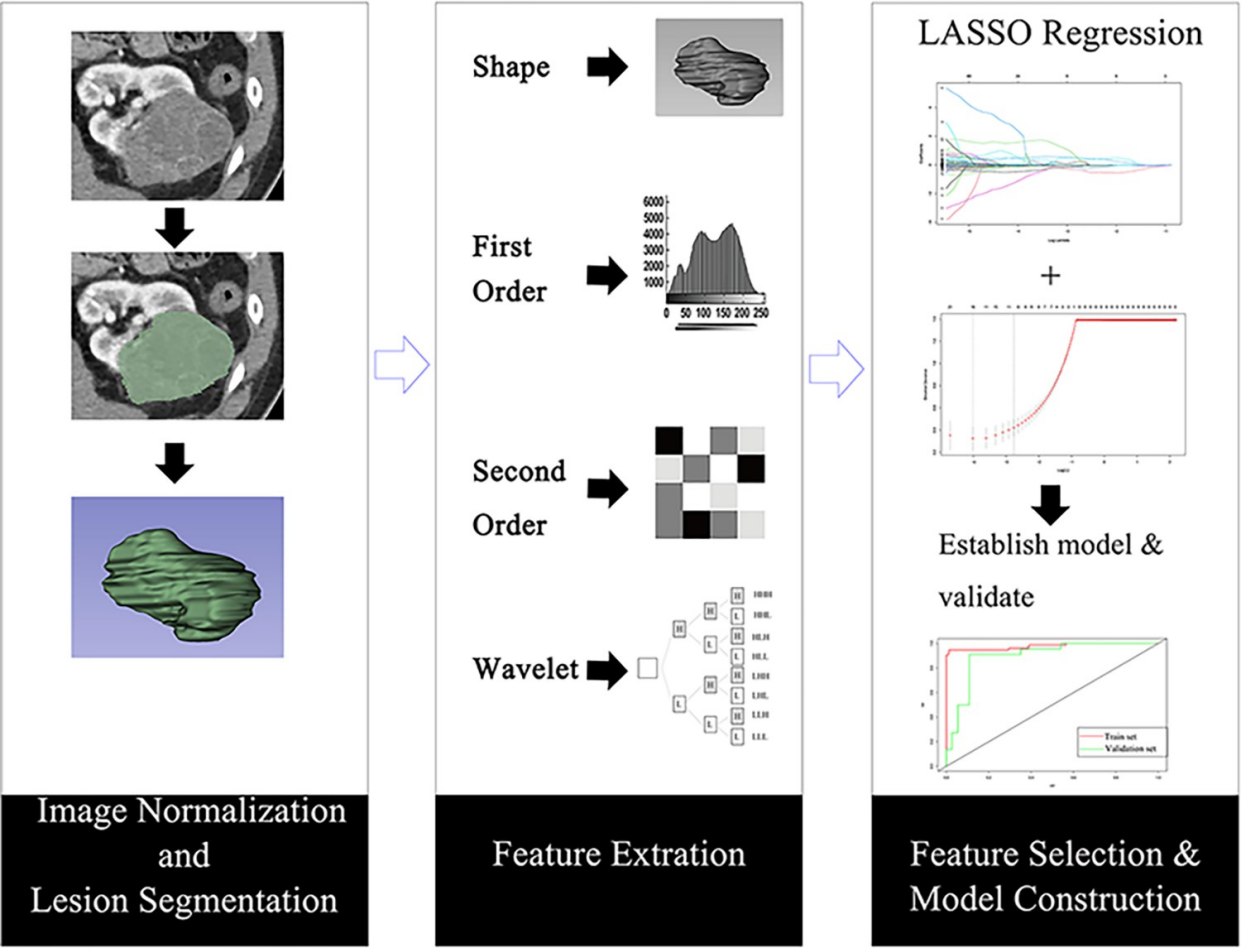
Workflow for extracting radiomics features.

The discrimination of the models for benign and malignant CRMs was evaluated using the area under the curve (AUC) of the Receiver Operating Characteristic (ROC) curve, and the sensitivity, specificity, and accuracy of diagnosis of benign and malignant CRMs by the models and radiologists were compared. (**Fig 1**).

## Results

### Patient characteristics

**Fig 2:**
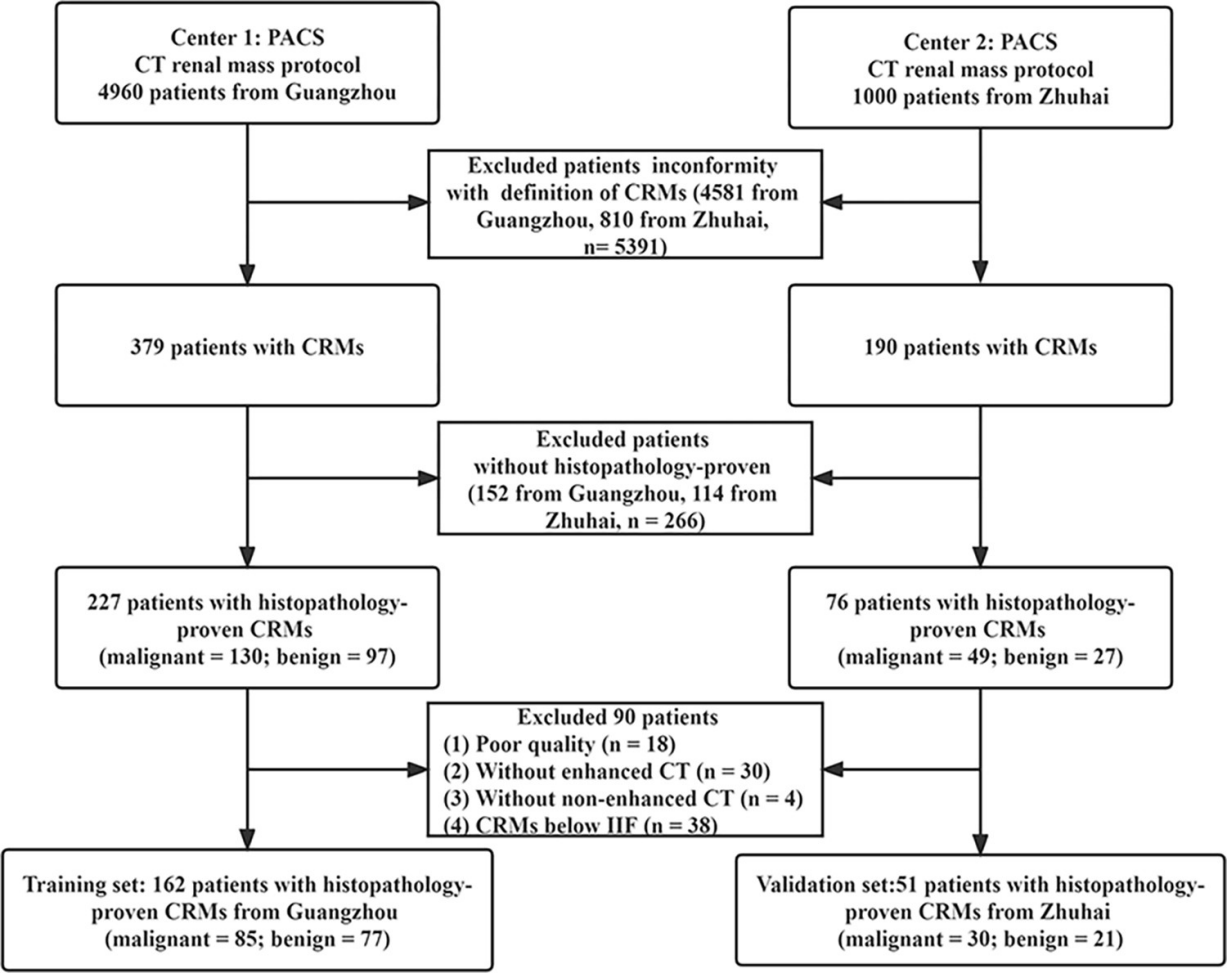
Flow chart of patient selection.

This study included 213 patients (108 males; 105 females; mean age, 58.8 ± 11.7 years) with CRMs. Of these 98 patients (54 males; 44 females; mean age, 57.8 ±14.0 years) had benign CRMs, and 115 patients (54 males; 61 females; mean age, 59.8 ±11.4 years) had malignant CRMs (**Fig 2**). There were no significant differences in age, sex, mass location and size between patients with benign and malignant CRMs (**Table 1**). All the benign CRMs were simple kidney cysts except one case of angiomyolipoma (AML). All the malignant CRMs were clear cell carcinoma except one case of mixed epithelial and stromal tumor of the kidney (MESTK).

**Table 1:** Demographic and clinical characteristics of the patients with CRMs.

Characteristics	Benign (n = 98)	Malignant (n = 115)	*P* value
Age (years), mean ± SD	57.8 ±14.0	59.8 ± 11.4	0.127
***Gender***			0.223
Male	54 [55.1%]	54 [41.9%]	
Female	44 [44.9%]	61 [58.1%]	
Mass size (cm), mean ± SD	4.80 ± 1.60	5.19 ± 1.69	0.546
***Location***			0.278
Right kidney	49 [50.0%]	66 [57.4%]	
Left kidney	49 [50.0%]	49 [42.6%]	
***Histology subtype***			<0.0001
Simple kidney cyst	97	0	
Clear cell carcinoma	0	114	
Other	1	1	
***Bosniak classification***			<0.0001
ⅡF	80 [81.6%]	19 [16.5%]	
Ⅲ	18 [18.5%]	31 [27.0%]	
Ⅳ	0 [0%]	65 [56.5%]	

### Diagnostic accuracy of the radiologists

On unenhanced CT images, the sensitivity, specificity and accuracy of the two radiologists for discriminating between benign and malignant CRMs were 80.0%, 94.6% and 86.5% and 84.3%, 91.3% and 87.0%, respectively. On AP CT images, the sensitivity, specificity and accuracy of the two radiologists for discriminating between benign and malignant CRMs were 95.6%, 100% and 97.1%, and 95.6%, 100% and 97.1%, respectively. On VP CT images, the sensitivity, specificity and accuracy of the two radiologists for discriminating between benign and malignant CRMs were 95.6%, 97.8% and 96.6% and 93.1%, 93.5% and 93.2%, respectively (**Table 2**).

**Table 2:** Sensitivity, specificity, accuracy and AUC of radiologists and radiomics models in the training and validation sets.

	Sensitivity (%)	Specificity (%)	Accuracy (%)	AUC	p value, vs. R ^1st^	p value, vs. R ^2nd^
***Non-enhanced***						
R ^1st^	80	94.6	86.5	N/A	N/A	N/A
R ^2nd^	84.3	91.3	87.0	N/A	N/A	N/A
Train Model	98.8	94.7	96.9	0.98	0.001**	0.003**
Validation Model	90.0	90.5	90.2	0.90	0.230	0.590
***AP***						
R ^1st^	95.6	100	97.1	N/A	N/A	N/A
R ^2nd^	95.6	100	97.1	N/A	N/A	N/A
Train Model	95.3	99.0	97.1	0.99	0.663	0.663
Validation Model	86.7	95.2	88.3	0.93	0.007**	0.007**
***VP***						
R ^1st^	95.6	97.8	96.6	N/A	N/A	N/A
R ^2nd^	93.1	93.5	93.2	N/A	N/A	N/A
Train Model	97.6	100	98.8	0.99	0.453	0.051
Validation Model	93.3	90.5	92.1	0.95	0.236	0.786

Note: AP, Arterial phase; VP, Venous phase; R, Radiologist

*, < 0.05

**, < 0.01

### Radiomics feature analysis

**Fig 3:**
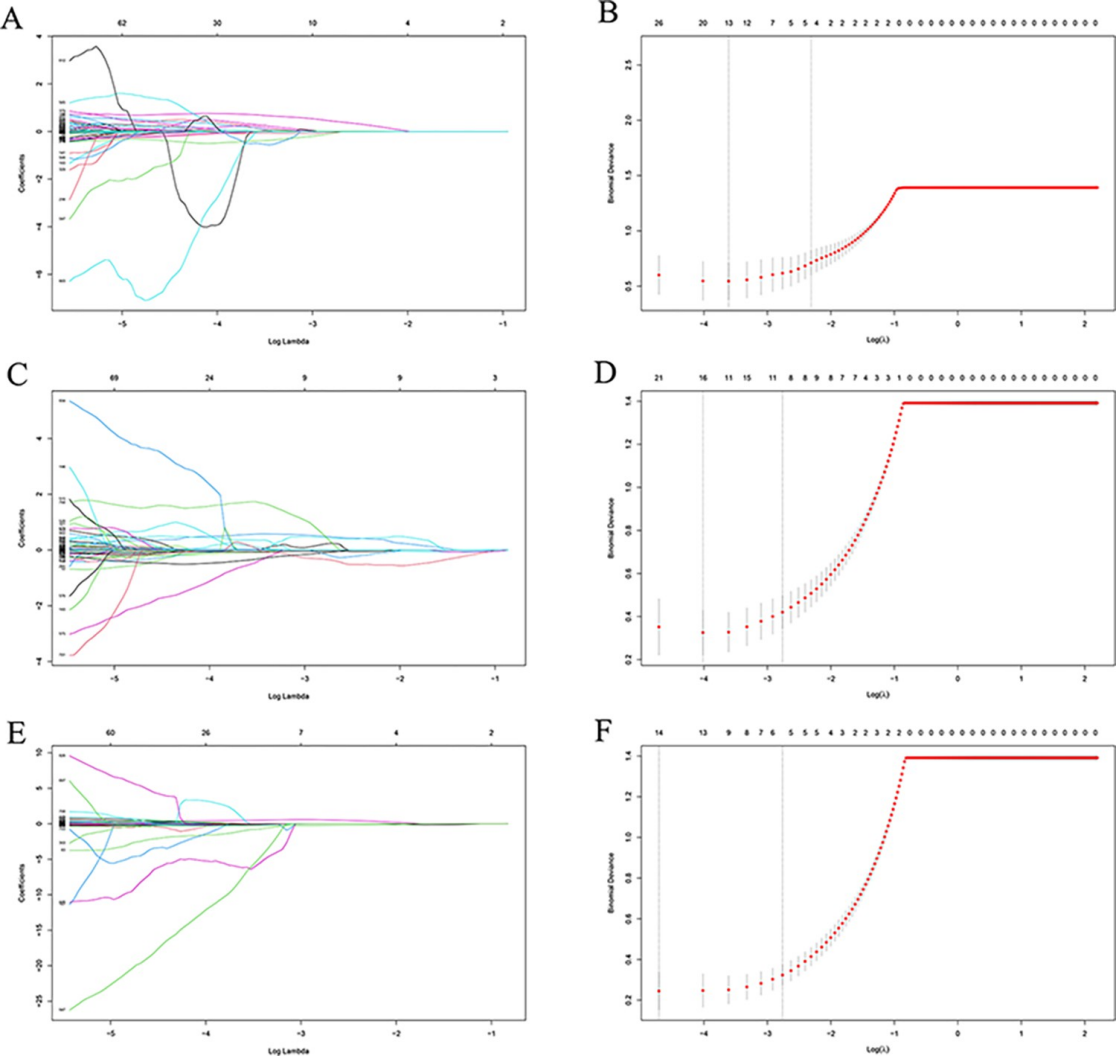
(A-F) Radiomics feature selection based on the least absolute shrinkage and selection operator (LASSO) and 10-fold cross-validation. LASSO coefficient profiles of the radiomics features (A, C, E). 4 features (B) on unenhanced CT images, 9 features (D) on AP CT images, and 5 features (F) on VP CT images were selected with the smallest binomial deviance.

After univariate analyses, 216, 164 and 181 stable radiomics features were extracted from unenhanced, AP and VP CT images, respectively, in the training set. The LASSO algorithm and 10-fold cross validation were used to select optimal subsets of radiomics features, which included 4 features on unenhanced CT images (**Fig 3A and 3B**), 5 features on AP CT images (**Fig 3E and 3F**), and 9 features on VP CT images (**Fig 3C and 3D**). Bidirectional elimination was used to further filter unrelated features and 4 features (Original_glcm_MaximumProbability, Wavelet.LHH_firstorder_Median, Wavelet.LLL_firstorder_90Percentile, Wavelet.LLL_firstorder_Median) on unenhanced CT images, 2 features (Wavelet.LLL_firstorder_Median, wavelet.LLL_firstorder_Uniformity) on AP CT images and 2 features (original_firstorder_Median, wavelet.LLL_gldm_DependenceEntropy) on VP CT images were extracted. Radiomics models were built using logistic regression and tested in the validation set.

### Diagnostic accuracy of the radiomics models

**Fig 4:**
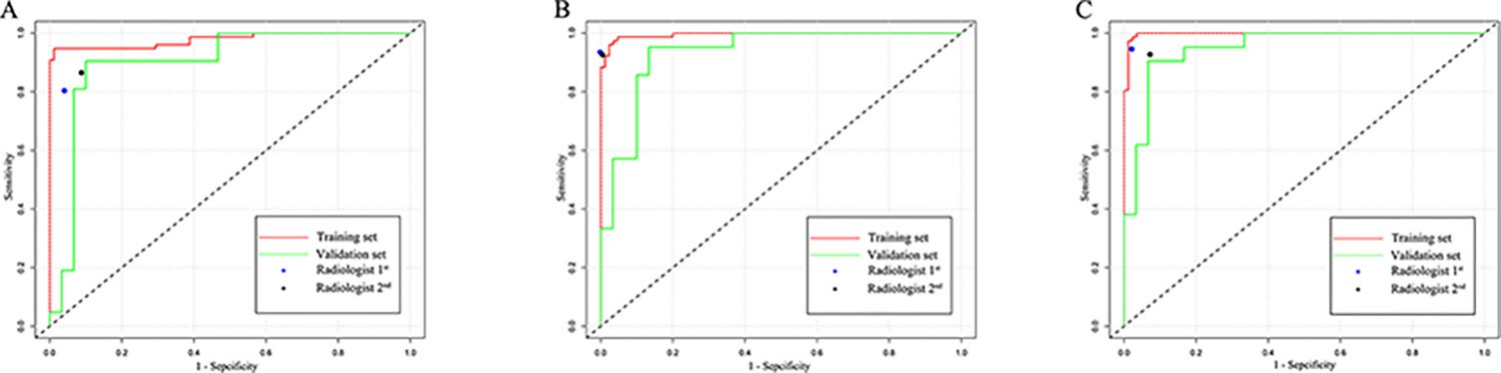
Performance evaluation of the models in the training and validation sets. Receiver operating characteristic curve of the radiomics models on unenhanced (A), AP (B) and VP (C) CT images.

On unenhanced CT images in the training set and validation set, the AUC, specificity, sensitivity and accuracy of the radiomics model for discriminating between benign and malignant CRMs were 98.0% (95%CI: 95.0–99.0%), 98.8%, 94.7% and 96.9% and 90.0 (95%CI: 81–98%), 90.0%, 90.5% and 90.2%, respectively (**Fig 4A**). On AP CT images in the training set and validation set, the AUC, specificity, sensitivity and accuracy of the radiomics model for discriminating between benign and malignant CRMs were 99.0% (95%CI: 95–100%), 95.3%, 99.0% and 97.1% and 93.0% (95%CI: 86–99%), 86.7%, 95.2% and 88.3%, respectively (**Fig 4B**). On VP CT images in the training set and validation set, the AUC, specificity, sensitivity and accuracy of the radiomics model for discriminating between benign and malignant CRMs were 99% (95%CI: 98%-1.00%), 97.6%, 100.0% and 98.8% and 95.0% (95%CI: 90%-100%), 93.3%, 90.5% and 92.1%, respectively (**Fig 4C**) (**Table 2**).

The AUC, specificity, sensitivity and accuracy of the radiomics models for discriminating between benign and malignant CRMs in the training set and validation set were not significantly different (**S1 Table**).

The AUC, specificity, sensitivity and accuracy for discriminating between benign and malignant CRMs differed significantly on unenhanced images in the training set vs. each radiologist (p = 0.001 and 0.003) but not in the validation set (p = 0.230 and 0.590); differed significantly on AP images in the validation set vs. each radiologist (p = 0.007 and 0.007) but not in the training set (p = 0.663 and 0.663); and there were no differences on VP images in the training or validation sets vs. each radiologist (training set: p = 0.453 and 0.051, validation set: p = 0.236 and 0.786).

## Discussion

This multicenter study used a radiomics approach to discriminate between benign and malignant CRMs on all phases of a triple phase CT protocol (unenhanced, AP and VP) and compared the diagnostic accuracy of the radiomics approach to experienced radiologists. Findings showed that diagnostic performance of the radiomics models on unenhanced, AP and VP CT images was satisfactory and similar to or better than experienced radiologists.

The Bosniak classification is used to guide management of CRMs; however, it may be inadequate. Terms such as “cystic”, “solid”, “walls” and “septa” may be ambiguous [18–23]. There is large inter-reader variability [24], ranging from 6–75% for Bosniak II, IIF, and III masses [8]. Most CRMs are found incidentally, and examinations may not be designed to apply the Bosniak classification [25]. The septa of a Bosniak IIF mass may display “perceived” but not “measurable” enhancement [1], which introduces subjectivity bias. The Bosniak classification cannot be applied to very small CRMs [26].

Compared to visual inspection of CT images for the diagnosis of CRMs, a radiomics approach may provide a more comprehensive representation of the microscopic heterogeneity of the masses [27–29], offering an accurate representation of a lesion’s pathology. The present study builds on prior research that verified the utility and stability of radiomics features for diagnosis of CRMs [10, 12, 15, 30], applies the radiomics approach independent of the Bosniak classification, and compares the performance of radiomics models with the Bosniak classification in pathology-proven benign and malignant CRMs. Although a previous study has demonstrated that a CT texture-based machine learning algorithm has the ability to differentiate benign from malignant CRMs on contrast-enhanced abdominal CT scans [15], to the authors’ knowledge, the present study is the first to apply a radiomics approach to CRM diagnosis on unenhanced CT images alone, and to compare the diagnostic performance of the radiomics models with experienced radiologists [15–17].

The radiomics models on unenhanced, AP and VP CT images in the validation set had relatively high sensitivity (86.7%–93.3%) and specificity (88.3%–92.1%) for distinguishing benign and malignant CRMs. The sensitivity, specificity and accuracy of the AP radiomics models in the validation set were lower than the radiologists; however, the sensitivity, specificity and accuracy of the unenhanced and VP radiomics models in the validation set were not significantly different compared to either radiologist. These data imply that the single phase radiomics models, especially the unenhanced and VP models, have important and practical clinical applications.

On unenhanced CT images, diagnostic accuracy of the two radiologists for discriminating between benign and malignant CRMs was lower compared to AP and VP images, possibly due to the poor contrast between normal and pathological tissue. The unenhanced radiomics model seemed to be less impact by the lower tissue contrast, and provided satisfactory diagnostic efficacy. These data imply that the unenhanced radiomics model has potential as a valuable diagnostic tool for CRMs in clinical and radiological practices. Most CRMs are found incidentally, for example, on chest unenhanced or annual CT examinations, or CT contrast agent may be contraindicated in patients with renal insufficiency making diagnosis of CRMs difficult. The unenhanced radiomics model may assist in screening, provide a preliminary diagnosis, and inform clinical decision-making.

This study was associated with several limitations. First, visual inspection of unenhanced, AP and VP CT images at 2-week intervals may have led to bias as radiologists may recall their initial diagnoses. Second, each center in this study used similar CT scanning systems and CT scanning parameters, so findings may not be generalizable to centers that use different systems and parameters. Third, the validation set included more malignant (n = 30) than benign (n = 21) CRMs, which may have introduced bias. Finally, our study did not apply other machine learning classifiers such as random forest, decision tree, or support vectors. These will be applied to strengthen our findings in future research.

## Conclusion

In conclusion, this study developed radiomics models that have clinical utility for discriminating benign and malignant CRMs on unenhanced and enhanced CT images. The performance of the radiomics model on unenhanced CT images was similar to experienced radiologists and may have value as a potential screening and diagnostic tool for CRMs.

## Supporting information

S1 Checklist *PLOS ONE* clinical studies checklist. (DOCX)

S2 Checklist STROBE statement—checklist of items that should be included in reports of observational studies. (DOCX)

S1 Table Comparison of radiomics models in the training and validation sets. (DOCX)

S1 Data (ZIP)

S2 Data (ZIP)

## References

[1] Silverman SG, Pedrosa I, Ellis JH, Hindman NM, Schieda N, Smith AD, et al. Bosniak Classification of Cystic Renal Masses, Version 2019: An Update Proposal and Needs Assessment. Radiology. 2019; 292(2):475–88. doi: 10.1148/radiol.2019182646 31210616 PMC6677285

[2] Terada N, Arai Y, Kinukawa N, Yoshimura K, Terai A. Risk factors for renal cysts. BJU Int. 2004; 93(9):1300–2. doi: 10.1111/j.1464-410X.2004.04844.x . 15180627

[3] Carrim ZI, Murchison JT. The prevalence of simple renal and hepatic cysts detected by spiral computed tomography. Clin Radiol. 2003; 58(8):626–9. doi: 10.1016/s0009-9260(03)00165-x . 12887956

[4] Silverman SG, Israel GM, Herts BR, Richie JP. Management of the incidental renal mass. Radiology. 2008; 249(1):16–31. doi: 10.1148/radiol.2491070783 . 18796665

[5] Go AS, Chertow GM, Fan D, McCulloch CE, Hsu CY. Chronic kidney disease and the risks of death, cardiovascular events, and hospitalization. N Engl J Med. 2004; 351(13):1296–305. doi: 10.1056/NEJMoa041031 . 15385656

[6] Sun M, Thuret R, Abdollah F, Lughezzani G, Schmitges J, Tian Z, et al. Age-adjusted incidence, mortality, and survival rates of stage-specific renal cell carcinoma in North America: a trend analysis. Eur Urol. 2011; 59(1):135–41. doi: 10.1016/j.eururo.2010.10.029 . 21035250

[7] Sun M, Trinh QD, Bianchi M, Hansen J, Hanna N, Abdollah F, et al. A non-cancer-related survival benefit is associated with partial nephrectomy. Eur Urol. 2012; 61(4):725–31. doi: 10.1016/j.eururo.2011.11.047 . 22172373

[8] Schoots IG, Zaccai K, Hunink MG, Verhagen P. Bosniak Classification for Complex Renal Cysts Reevaluated: A Systematic Review. J Urol. 2017; 198(1):12–21. doi: 10.1016/j.juro.2016.09.160 . 28286071

[9] Gillies RJ, Kinahan PE, Hricak H. Radiomics: Images Are More than Pictures, They Are Data. Radiology. 2016; 278(2):563–77. doi: 10.1148/radiol.2015151169 . 26579733 PMC4734157

[10] Lubner MG. Radiomics and Artificial Intelligence for Renal Mass Characterization. Radiol Clin North Am. 2020; 58(5):995–1008. doi: 10.1016/j.rcl.2020.06.001 . 32792129

[11] Mühlbauer J, Egen L, Kowalewski K-F, Grilli M, Walach MT, Westhoff N, et al. Radiomics in Renal Cell Carcinoma—A Systematic Review and Meta-Analysis. Cancers. 2021; 13(6). doi: 10.3390/cancers13061348 33802699 PMC8002585

[12] Ursprung S, Beer L, Bruining A, Woitek R, Stewart GD, Gallagher FA, et al. Radiomics of computed tomography and magnetic resonance imaging in renal cell carcinoma-a systematic review and meta-analysis. Eur Radiol. 2020; 30(6):3558–66. doi: 10.1007/s00330-020-06666-3 . 32060715 PMC7248043

[13] Li S, Liu J, Xiong Y, Han Y, Pang P, Luo P, et al. Application Values of 2D and 3D Radiomics Models Based on CT Plain Scan in Differentiating Benign from Malignant Ovarian Tumors. Biomed Res Int. 2022; 2022:5952296. doi: 10.1155/2022/5952296 . 35224097 PMC8872698

[14] Yang X, He J, Wang J, Li W, Liu C, Gao D, et al. CT-based radiomics signature for differentiating solitary granulomatous nodules from solid lung adenocarcinoma. Lung Cancer. 2018; 125:109–14. doi: 10.1016/j.lungcan.2018.09.013 . 30429007

[15] Miskin N, Qin L, Silverman SG, Shinagare AB. Differentiating Benign From Malignant Cystic Renal Masses: A Feasibility Study of Computed Tomography Texture-Based Machine Learning Algorithms. J Comput Assist Tomogr. 2023. doi: 10.1097/RCT.0000000000001433 . 37184999

[16] Dana J, Lefebvre TL, Savadjiev P, Bodard S, Gauvin S, Bhatnagar SR, et al. Malignancy risk stratification of cystic renal lesions based on a contrast-enhanced CT-based machine learning model and a clinical decision algorithm. Eur Radiol. 2022; 32(6):4116–27. doi: 10.1007/s00330-021-08449-w . 35066631

[17] He QH, Feng JJ, Lv FJ, Jiang Q, Xiao MZ. Deep learning and radiomic feature-based blending ensemble classifier for malignancy risk prediction in cystic renal lesions. Insights Imaging. 2023; 14(1):6. doi: 10.1186/s13244-022-01349-7 . 36629980 PMC9834471

[18] Corica FA, Iczkowski KA, Cheng L, Zincke H, Blute ML, Wendel A, et al. Cystic renal cell carcinoma is cured by resection: a study of 24 cases with long-term followup. J Urol. 1999; 161(2):408–11. doi: 10.1016/s0022-5347(01)61903-7 . 9915413

[19] Webster WS, Thompson RH, Cheville JC, Lohse CM, Blute ML, Leibovich BC. Surgical Resection Provides Excellent Outcomes for Patients With Cystic Clear Cell Renal Cell Carcinoma. Urology. 2007; 70(5):900–04. doi: 10.1016/j.urology.2007.05.029 18068445

[20] Jhaveri K, Gupta P, Elmi A, Flor L, Moshonov H, Evans A, et al. Cystic renal cell carcinomas: do they grow, metastasize, or recur? AJR Am J Roentgenol. 2013; 201(2):W292–6. doi: 10.2214/AJR.12.9414 . 23883243

[21] Cooperberg MR, Mallin K, Kane CJ, Carroll PR. Treatment trends for stage I renal cell carcinoma. J Urol. 2011; 186(2):394–9. doi: 10.1016/j.juro.2011.03.130 . 21679982

[22] Daskivich TJ, Tan H-J, Litwin MS, Hu JC. Life Expectancy and Variation in Treatment for Early Stage Kidney Cancer. Journal of Urology. 2016; 196(3):672–77. doi: 10.1016/j.juro.2016.03.133 27012644

[23] Kane CJ, Mallin K, Ritchey J, Cooperberg MR, Carroll PR. Renal cell cancer stage migration: analysis of the National Cancer Data Base. Cancer. 2008; 113(1):78–83. doi: 10.1002/cncr.23518 . 18491376

[24] Siegel CL, McFarland EG, Brink JA, Fisher AJ, Humphrey P, Heiken JP. CT of cystic renal masses: analysis of diagnostic performance and interobserver variation. American Journal of Roentgenology. 1997; 169(3):813–18. doi: 10.2214/ajr.169.3.9275902 9275902

[25] Silverman SG, Israel GM, Trinh Q-D. Incompletely Characterized Incidental Renal Masses: Emerging Data Support Conservative Management. Radiology. 2015; 275(1):28–42. doi: 10.1148/radiol.14141144 25799334

[26] Patel J, Davenport MS, Khalatbari S, Cohan RH, Ellis JH, Platt JF. In Vivo Predictors of Renal Cyst Pseudoenhancement at 120 kVp. American Journal of Roentgenology. 2014; 202(2):336–42. doi: 10.2214/AJR.13.10915 24450674 PMC4328313

[27] Wang JC, Fu R, Tao XW, Mao YF, Wang F, Zhang ZC, et al. A radiomics-based model on non-contrast CT for predicting cirrhosis: make the most of image data. Biomark Res. 2020; 8:47. doi: 10.1186/s40364-020-00219-y . 32963787 PMC7499912

[28] Wang J, Tang S, Mao Y, Wu J, Xu S, Yue Q, et al. Radiomics analysis of contrast-enhanced CT for staging liver fibrosis: an update for image biomarker. Hepatol Int. 2022; 16(3):627–39. doi: 10.1007/s12072-022-10326-7 . 35347597 PMC9174317

[29] Mao Y, Wang J, Zhu Y, Chen J, Mao L, Kong W, et al. Gd-EOB-DTPA-enhanced MRI radiomic features for predicting histological grade of hepatocellular carcinoma. Hepatobiliary Surg Nutr. 2022; 11(1):13–24. doi: 10.21037/hbsn-19-870 35284527 PMC8847875

[30] Konik A, Miskin N, Guo Y, Shinagare AB, Qin L. Robustness and performance of radiomic features in diagnosing cystic renal masses. Abdom Radiol (NY). 2021; 46(11):5260–67. doi: 10.1007/s00261-021-03241-2 . 34379150

