# Correction: Enhanced and unenhanced: Radiomics models for discriminating between benign and malignant cystic renal masses on CT images: A multi-center study

**DOI:** 10.1371/journal.pone.0315835

**Published:** 2024-12-11

**Authors:** Lesheng Huang, Wenhui Feng, Wenxiang Lin, Jun Chen, Se Peng, Xiaohua Du, Xiaodan Li, Tianzhu Liu, Yongsong Ye

There are errors in the author affiliations. The correct affiliations are as follows:

Lesheng Huang^1^, Wenhui Feng^2^, Wenxiang Lin^2^, Jun Chen^1^, Se Peng^3^, Xiaohua Du^4^, Xiaodan Li ^5^, Tianzhu Liu^1^*, Yongsong Ye^6^

**1** Department of Radiology, Guangdong Provincial Hospital of Traditional Chinese Medicine, Zhuhai, China, **2** Department of Radiology, Zhuhai People’s Hospital, Zhuhai, China, **3** Department of Laboratory, Guangdong Provincial Hospital of Traditional Chinese Medicine, Zhuhai, China, **4** Department of Pathology, Guangdong Provincial Hospital of Traditional Chinese Medicine, Guangzhou, China, **5** Department of Gynaecology, Guangdong Provincial Hospital of Traditional Chinese Medicine, Zhuhai, China, **6** Department of Radiology, Guangdong Provincial Hospital of Traditional Chinese Medicine, Guangzhou, China
