# Supplementary material for: Enhanced and unenhanced: Radiomics models for discriminating between benign and malignant cystic renal masses on CT images: A multi-center study
Source: PLoS One. 2023 Sep 28;18(9):e0292110. doi: 10.1371/journal.pone.0292110 (PMC10538730; doi:10.1371/journal.pone.0292110)
Supplement: S1 Table — (DOCX) [file pone.0292110.s003.docx]

**Table S1** Comparison of radiomics models in the training and validation sets.

|  | U vs. AP | U vs. VP | AP vs. VP |
| --- | --- | --- | --- |
| *p* value training set | 0.294 | 0.217 | 0.637 |
| *p* value validation set | 0.759 | 0.912 | 0.837 |

U, unenhanced; AP, Arterial phase; VP, Venous phase
